# *Francisella tularensis novicida* infection competence differs in cell lines derived from United States populations of *Dermacentor andersoni and Ixodes scapularis*

**DOI:** 10.1038/s41598-018-30419-4

**Published:** 2018-08-23

**Authors:** Kathryn E. Reif, Jessica K. Ujczo, Debra C. Alperin, Susan M. Noh

**Affiliations:** 1 Animal Disease Research Unit, Agriculture Research Service, US Department of Agriculture, Pullman, Washington USA; 2 Department of Veterinary Microbiology and Pathology, Washington State University, Pullman, Washington USA

## Abstract

In the United States, *Dermacentor spp*. are common vectors of *Francisella tularensis* subspecies (ssp.), while *Ixodes scapularis* is not, though the geographic distribution and host range of pathogen and tick overlap. To examine if differences in infection competence at the cellular level underpin these ecological differences, we evaluated the competence of *D. andersoni* (DAE100) and *I. scapularis* (ISE6) cell lines to support *F. tularensis* ssp. *novicida* (*F. novicida*) infection. Importantly, *D. andersoni* is a vector for both *F. tularensis* spp. *tularensis*, and *F. novicida*. We hypothesized *F. novicida* infection would be more productive in *D. andersoni* than in *I. scapularis* cells. Specifically, we determined if there are differences in *F. novicida* i) invasion, ii) replication, or iii) tick cell viability between DAE100 and ISE6 cells. We further examined the influence of temperature on infection kinetics. Both cell lines were permissive to *F. novicida* infection; however, there were significantly higher bacterial levels and mortality in DAE100 compared to ISE6 cells. Infection at environmental temperatures prolonged the time bacteria were maintained at high levels and reduced tick cell mortality in both cell lines. Identifying cellular determinants of vector competence is essential in understanding tick-borne disease ecology and designing effective intervention strategies.

## Introduction

Tick-borne diseases are the most common vector-borne diseases of humans in the United States with the number of reported cases steadily increasing and the distribution of tick vector species and tick-borne pathogens continuing to expand and overlap. In the United States, the number of reported cases of tick-borne disease increased from ~17,000 cases in 2001 to >40,000 cases in 2014^1^. However, due to under-reporting, the actual number of cases in the United States is estimated to be >400,000 per year^2^. There are multiple reasons for the increase in tick-borne diseases including expansion of tick geographic ranges, broadening of tick-borne disease endemic regions, over-abundance of wildlife populations that support ticks, climate changes, and improved diagnostics and surveillance^3,4^. However, the foundation of tick-borne disease epidemiology is vector competence, which is the ability of the vector to acquire, maintain, and transmit a pathogen.

Vector competence for a given tick-borne pathogen can vary among different tick species and within populations of the same tick species^5,6^. Moreover, vector competence can be influenced by numerous biotic and abiotic variables. Examples of biotic variables that can affect vector competence include the presence of host cell receptors for pathogen attachment and entry, accessibility to required nutrients, an innate immune system that allows pathogen replication and, direct or indirect interaction with co-infecting microbiota. Examples of abiotic variables that can affect vector competence, and more broadly vectorial capacity, include temperature and humidity.

With the exception of *Borrelia burgdorferi*, an extracellular spirochete, the determinants of vector competence for tick-borne pathogens, particularly at the cellular and molecular level, are largely unknown. Most other significant bacterial, tick-borne pathogens of humans, including *Rickettsia rickettsii*, *Francisella tularensis*, and *Anaplasma phagocytophilum* are intracellular pathogens and the determinants of vector competence for these pathogens are likely to be significantly different from *B. burgdorferi*. For intracellular tick-borne bacterial pathogens, limited experimental models and genetic tools have hampered identification of cellular and molecular determinants of vector competence^7^.

In the northeastern United States, *F. tularensis* ssp. is maintained by rabbits and *Dermacentor* spp. ticks. *Ixodes scapularis* is present in these regions and feeds on rabbits; however, *I. scapularis* is not recognized as a vector of *F. tularensis* spp. in this region^8^. Using cell lines derived from *D. andersoni* (DAE100) and *I. scapularis* (ISE6), we investigated if the ecological relevance of these tick species in the transmission of *F. tularensis* ssp. was mirrored at a cellular level. *F. tularensis* ssp. *novicida* (*F. novicida*), used in these experiments, is not a human pathogen and little is known regarding its epidemiology^9^. However, within *Dermacentor* spp. ticks, *F. novicida* and *F. tularensis* spp. *tularensis*, have similar lifecycles, including transmission^10,11^. Thus *F. novicida* serves as non-hazardous laboratory model for *F. tularensis* spp. *tularensis* in ticks.

We hypothesized that *F. novicida* would infect both tick cell lines but would establish a more productive infection in the cell line derived from *D. andersoni*. We also hypothesized that abiotic variables such as temperature would additionally affect infection kinetics. To address our hypotheses, we determined the following: i) the competence of DAE100 and ISE6 to support *F. novicida* infection and replication; ii) the impact of infection on tick cell viability; and, iii) infection kinetic differences at tick blood-feeding versus environmental temperatures. We present the results of our study in the context of how these *in vitro* assays can be used to identify determinants of vector competence for intracellular tick-borne bacterial pathogens.

## Results

### *F. novicida*-infection kinetics differ during infection of DAE100 and ISE6 tick cells

Using *F. novicida* as a model to examine if the ecological relevance of *D. andersoni* and *I. scapularis* for *F. tularensis* ssp. transmission is mirrored at the cellular level, we compared the competence of the DAE100 and the ISE6 cell lines to become infected with and support *F. novicida* replication. Tick cell cultures were inoculated with *F. novicida* and bacterial infection levels were measured at defined time points to determine cell line infection competence and bacterial infection kinetics. For the purpose of these experiments, bacterial infection kinetics is defined as the change in bacterial counts over time and is used as an indicator of successful bacterial replication in a given host cell. The tick cells were infected with *F. novicida* at an MOI of 100 and bacteria allowed to infect tick cells for two hours after which gentamicin pressure was maintained for the remainder of the experiment to prevent extracellular bacterial replication and tick cell reinfection.

**Figure 1:**
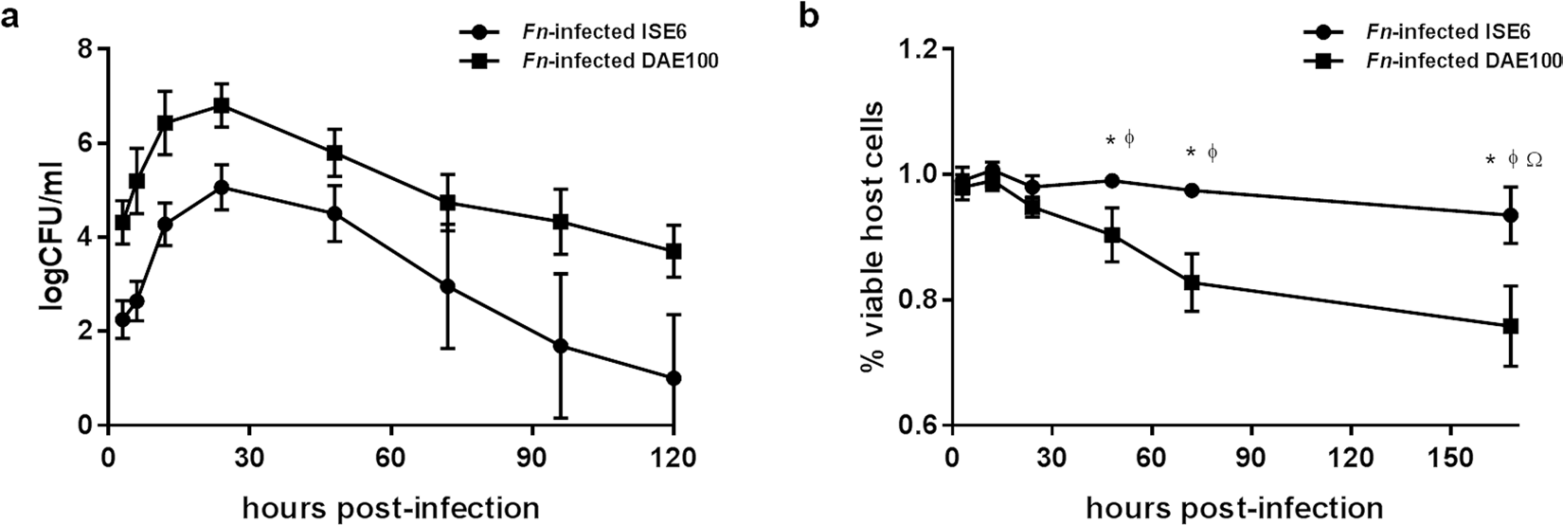
*F. novicida* infection kinetics in DAE100 and ISE6 cells. (**a**) Mean *F. novicida* bacterial levels (logCFU/ml) in tick cells over time. At all time points, *F. novicida* levels are significantly greater in DAE100 compared to ISE6 cultures. Presented data is the mean of six independent experiments, with each experiment performed in duplicate. Error bars represent SEM. (**b**) Tick cell viability (assessed using a trypan blue assay) during *F. novicida* infection was quantified by comparing tick cell viability at 12-, 24-, 48-, 72- and 168-hpi to 3-hpi. A two-way ANOVA and Bonferroni’s correction for multiple comparisons was used to compare viability data between cell lines (*represents significant difference between *F. novicida-infected* cell lines). A one-way ANOVA with Dunnett’s correction for multiple comparisons was used to compare viability at different time points within a cell line (^ɸ^represents significant difference in DAE100 cell viability from 3-hpi; ^Ω^represents significant difference in ISE6 cell viability from 3-hpi). Data were considered significant with p < 0.05.

Both DAE100 and ISE6 cells were infected and able to support *F. novicida* replication as indicated by increases in bacterial levels (Fig. 1a). Although both tick cell lines supported *F. novicida* infection, bacterial levels differed between cell lines. At all time-points, bacterial levels were on average 2.1 logs greater (*p* = <0.0001) in DAE100 cells, compared to ISE6 cells (Fig. 1a). In DAE100 cells, *F. novicida* infection level increased 309-fold from a mean of 2.1 × 10^4^ CFU/ml at 3 hours post-infection (hpi) to a mean peak of 6.4 × 10^6^ CFU/ml at 24 hpi. In ISE6 cells, *F. novicida* infection level increased 656-fold from a mean of 1.8 × 10^2^ CFU/ml at 3 hpi to a mean peak of 1.2 × 10^5^ CFU/ml at 24 hpi. Although the magnitude of *F. novicida* infection was significantly different in DAE100 and ISE6 cells, bacterial infection kinetics were similar at a culture level. Using linear regression, the slope of the best fit line for the *F. novicida* infection level between 3- and 24-hpi was calculated for each cell line. The overall slope of each line was not significantly different (slope for *F. novicida*-infected ISE6 = 0.1367 ± 0.0313 and slope for *F. novcida*-infected DAE100 = 0.111 ± 0.03773). In both tick cell lines, within 24 h after peak bacterial levels, bacterial infection levels were significantly lower and continued to significantly decrease at all subsequent time points. Together these data indicate that more *F. novicida* initially enter and replicate to greater levels in the cell line derived from *D. andersoni* compared to *I. scapularis*.

### DAE100 cell numbers are reduced during *F. novicida* infection

Vector competence can be affected by both pathogen virulence and inability of the pathogen to persist within the tick. For continued transmission, tick-borne pathogens must find a balance between persisting but not killing or being cleared by the vector. We previously demonstrated that *F. novicida* is able to infect, persist and be transmitted by *D. andersoni*, with no noticeable negative fitness effect on the ticks^10^. To determine the effect of *F. novicida* infection on tick cell viability, *F. novicida*-exposed tick cell cultures were stained with trypan blue. All tick cell viability counts were normalized to the number of viable tick cells present at 3-hpi and compared within and between tick cell lines at multiple time points. Tick cell death was significantly greater in DAE100 compared to ISE6 cells (Fig. 1b). In DAE100 cells, the first significant decline in tick cell number occurred at 48-hpi, following peak bacterial levels at 24-hpi. By 7 dpi (168-hpi), *F. novicida* infection resulted in a loss of approximately 25% of DAE100 cells. A significant decline in ISE6 cell numbers occurred only at 7 dpi, well after infection was almost completely resolved, indicating the decline in ISE6 numbers may be due to culture conditions rather than bacterial-mediated causes. This is supported by bacterial levels near the limit of detection at 120-hpi. In uninfected tick cells, the DAE100 cell count did not change over the experimental time course; however, the ISE6 cell count significantly increased by 3% by the end of the experiment (Supp Fig. 1). The minimal mortality in the *I. scapularis* cells compared to the *D. andersoni* cells could be due to the significantly lower infection levels and possibly more efficient detection and clearance of the pathogen.

### Environmental temperature mitigates tick cell mortality and supports retention of *F. novicida*-infected cells

Ticks are ectothermic and must adapt to different temperatures when they are ‘off-host’ in the environment or ‘on-host’ blood feeding. Temperature is a common abiotic factor and can affect pathogen infection kinetics. We initially studied *F. novicida* infection kinetics in tick cells at 34 °C, a temperature reflective of pathogen acquisition during tick blood feeding (and the normal culture temperature for these cell lines); however, for the majority of their lives, ticks are ‘off-host’ and persist at environmental temperatures. Therefore, tick-borne pathogens that rely on transstadial or transovarial maintenance for transmission, like many intracellular tick-borne bacterial pathogens, need to co-adapt with the tick vector to environmental temperatures. To determine whether temperature alters *F. novicida* infection kinetics we repeated the above assays at 24 °C, a representative environmental temperature. Additionally, we compared tick cell viability in uninfected and *F. novicida*-infected cells at ‘off-host’ environmental and ‘on-host’ blood feeding tick temperatures.

**Figure 2:**
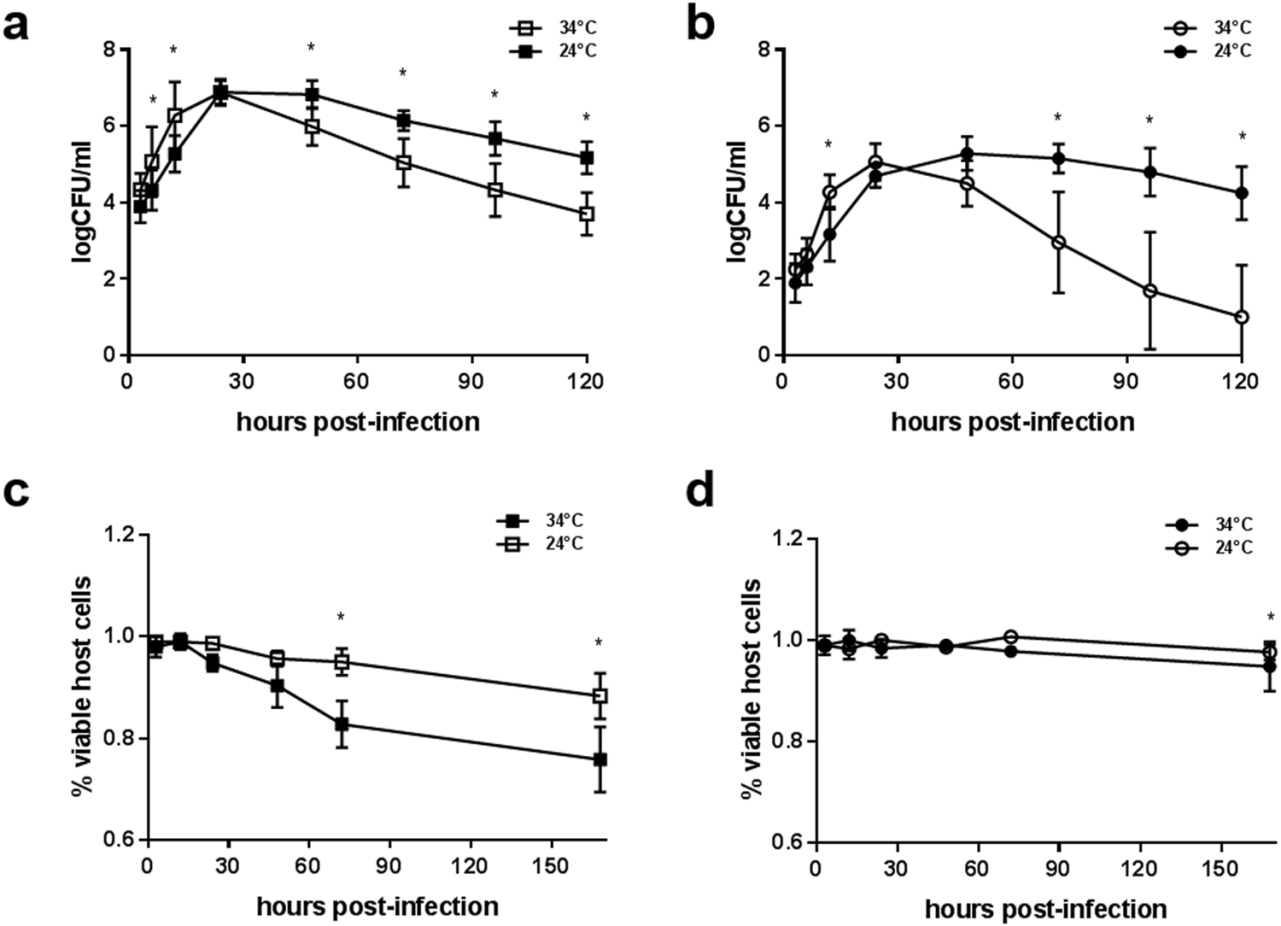
Temperature-induced changes in *F. novicida* infection kinetics. Comparison of mean *F. novicida* bacterial levels (logCFU/ml) in (**a**) DAE100 and (**b**) ISE6 cells over time at 34 °C and 24 °C. Presented data is the mean of six independent experiments, with each experiment performed in duplicate (*represents significant difference between *F. novicida-infected* cell lines). Comparison of (**c**) DAE100 and (**d**) ISE6 cell viability over time at 34 °C and 24 °C. Tick cell viability (assessed using a trypan blue assay) during *F. novicida* infection was quantified by comparing tick cell viability at 12-, 24-, 48-, 72- and 168-hpi to 3-hpi (*represents significant difference between *F. novicida-infected* cell lines). A two-way ANOVA and Bonferroni’s correction for multiple comparisons was used to compare log(CFU/ml) and viability data between cell lines. A one-way ANOVA with Dunnett’s correction for multiple comparisons was used to compare viability at different time points within a cell line. Error bars represent SEM. Data were considered significant with p < 0.05.

Incubation at an environmental temperature did not delay the time to peak infection in DAE100 cells (Fig. 2a). For ISE6 cells, peak infection did not occur until 48-hpi, but was not significantly different to cultures maintained at 34 °C with regard to bacterial levels (Fig. 2b). In both cell lines, the mean peak bacterial level did not change whether the assays were performed at 34 °C or 24 °C. Interestingly, in both tick cell lines, the decreased temperature resulted in extended elevated bacterial levels compared to the ‘on-host’ blood feeding temperature. In *F. novicida*-infected DAE100 cells incubated at 24 °C, elevated bacterial levels were maintained for an extended period and with significantly less tick cell mortality compared to 34 °C assays (Fig. 2c). Again, bacterial infection kinetics were similar at a culture level for each cell line at 24 °C, with the slope of the best fit line for the *F. novicida* infection level between 3- and 24-hpi not significantly different (slope for *F. novicida*-infected ISE6 = 0.1334 ± 0.0026 and slope for *F. novicida*-infected DAE100 = 0.1433 ± 0.0054). For a given cell line, no significant difference was observed in the slope of the best fit line for the *F. novicida* infection level between 3- and 24-hpi at a culture level based on incubation temperature (slope for *F. novicida*-infected ISE6 at 34 °C = 0.1367 ± 0.0314 versus at 24 °C = 0.1334 ± 0.0024; and, slope for *F. novicida*-infected DAE100 at 34 °C = 0.1163 ± 0.0305 versus at 24 °C = 0.1429 ± 0.0038).

The mean mortality for DAE100 cells was ~10% at 24 °C compared to ~25% at 34 °C. There was no significant tick cell mortality at any time point during *F. novicida*-infection of ISE6 cells at 24 °C (Fig. 2d). These results indicate that temperature does affect *F. novicida* infection kinetics in tick cells, where environmental temperatures are positively associated with tick cell survival.

In uninfected DAE100 cells, incubation at 24 °C had no effect on tick cell numbers, as a similar number of cells were present at 7-days post mock infection (Supp Fig. 2). For uninfected ISE6 cells, replication was stunted by incubation at 24 °C and cell counts did not significantly increase as observed at 34 °C (Supp Fig. 2). Therefore, the reduction in tick cell mortality was not a product of greater tick cell replication at lower temperatures, as uninfected tick cell counts did not increase over the experimental period for DAE100 cells.

### *F. novicida* infection rate and level are greater in DAE100 compared to ISE6 cells

**Figure 3:**
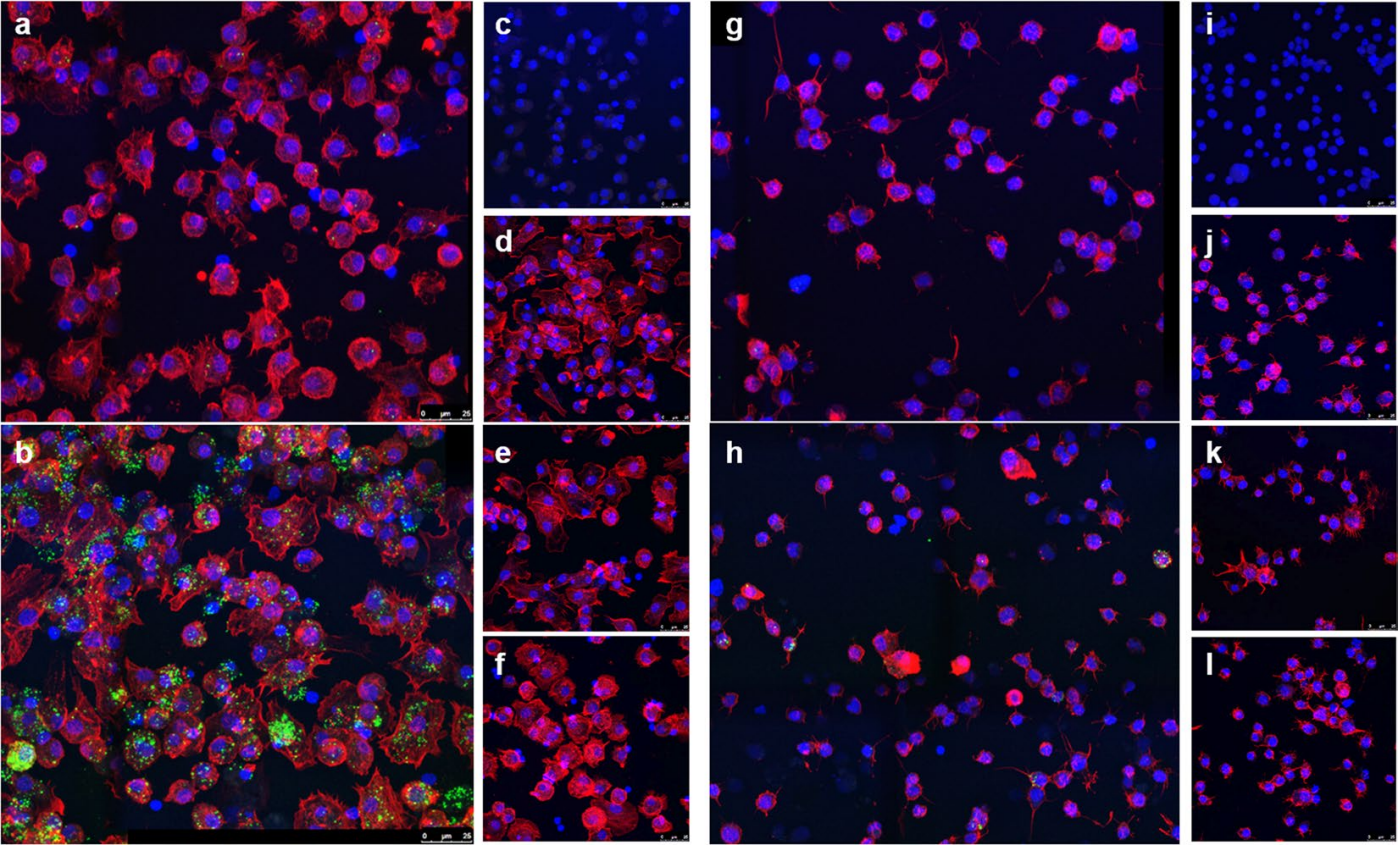
Visualization of *F. novicida*-infected DAE100 and ISE6 cells by confocal microscopy. Representative IFA confocal photomicrographs of *F. novicida*-infected DAE100 cells at (**a**) 3-hpi and (**b**) 18-hpi. Representative IFA confocal micrographs of *F. novicida*-infected ISE6 cells at (**g**) 3-hpi and 18-hpi. Staining controls for were performed for each cell type and include: (**c** and **i**) no primary antibody; (**d** and **j**) no secondary antibody; (**e** and **k**) irrelevant isotype-matched primary antibody; (**f** and **l**) uninfected tick cells.

**Figure 4:**
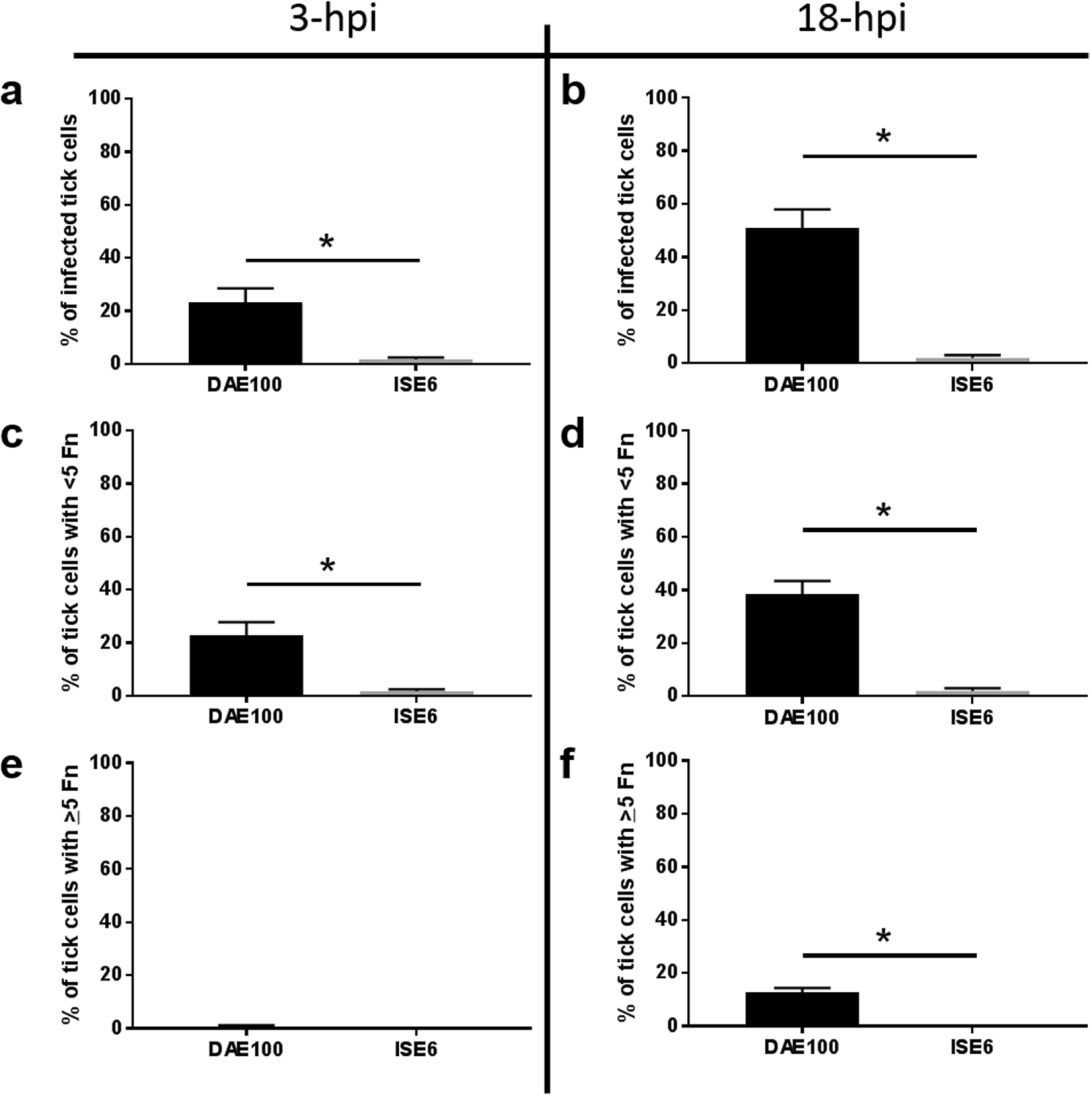
Infection of tick cells by *F. novicida*. Mean percent of *F. novicida*-infected tick cells at (**a**) 3-hpi and (**b**) 18-hpi. Mean percent of tick cells infected with <5 bacteria/cell at (**c**) 3-hpi and (**d**) 18-hpi. Mean percent of tick cells infected with >5 bacteria/cell at (**e**) 3-hpi and (**f**) 18-hpi. Presented data is the mean of four independent experiments, with each experimental mean derived from assessing 6 images/experiment (a minimum of 300 cells). Error bars represent SEM. The asterisk represents significant difference between the mean percent of *F. novicida*-infected cells at the indicated time points. An unpaired, two-tailed t test with Welch’s correction was used to analyze this data. Data were considered significant with p < 0.05.

In the previous experiments we found that *F. novicida* could infect cell lines derived from both *Dermacentor* and *Ixodes* tick species; however, the magnitude of infection in each of cell line was significantly different (Fig. 1). The vector competence of a given tick species can be influenced by insufficient bacterial infection or clearance by the host cell. We next wanted to address whether the observed differences between these cell lines were due to differences during infection at the individual cell level. To evaluate *F. novicida* infection in individual tick cells we used an immunofluorescence assay (IFA). Tick cells were visualized using DAPI for nuclear staining and phalloidin to stain actin to help identify individual cells and cell boundaries (Fig. 3). The *F. novicida* bacterial levels in individual DAE100 and ISE6 cells were quantified at 3-hpi and 18-hpi. At both time points, significantly more DAE100 cells were infected compared to ISE6 cells (Fig. 4a and b). Interestingly, despite maintenance of gentamicin pressure throughout the assay, *F. novicida*-infection rate significantly increased in DAE100 cultures from 23% at 3 hpi to 51% at 18 hpi. This increase in infection rate is likely due to cell-to-cell spread as is known to occur in *Francisella tularensis* and rickettsial pathogens^12^. To verify that the gentamicin was working as expected, we inoculated *F. novicida* directly into media containing the same lot and concentration of gentamicin used for the infection assays and serially diluted and plated the culture at 3-hpi. There was no bacterial growth, indicating the effectiveness of gentamicin in killing *F. novicida*. No difference in the *F. novicida*-infection rate was observed in ISE6 cells between 3 and 18 hpi. The absence of a significant infection rate of ISE6 cells at 18-hpi versus 3-hpi, also aids to demonstrate that the gentamicin effectively controlled extracellular bacterial replication and further tick cell infections.

The relative bacterial burden in individual cells was compared between DAE100 and ISE6 cells at 3- and 18-hpi. We quantified the number of infected cells that had <5 or >5 bacteria per cell to determine whether the difference in bacterial burden at the culture level was due to greater replication in DAE100 compared to ISE6 cells. Bacterial levels increased in individual DAE100 cells as the number of individual cells with >5 bacteria increased significantly from 3-hpi to 18-hpi (Fig. 4e and f). Moreover, as described above, and despite maintenance of gentamicin pressure, the percent of cells with <5 bacteria/cell also increased significantly between 3-hpi to 18-hpi (Fig. 4c and d). In total, the IFA results indicate that *F. novicida* is better able to enter and replicate in DAE100 cells compared to ISE6 cells. Interestingly, as reported for other cell lines and pathogens, there is spread of infection to closely neighboring cells as the mean percent of infected DAE100 cells increases over time despite maintenance of gentamicin pressure^12^.

### DAE100 are more phagocytic than ISE6 cells

**Figure 5:**
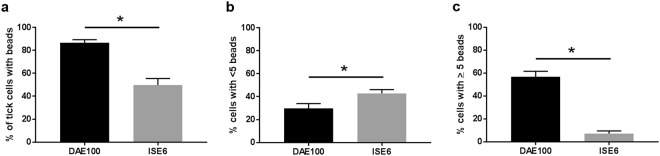
Internalization of beads by tick cells. (**a**) Mean percent of tick cells that internalized beads. (**b**) Mean percent of tick cells that internalized <5 beads/cell. (**c**) Mean percent of tick cells that internalized >5 beads/cell. Presented data is the mean of four independent experiments, with each experimental mean derived from assessing 6 images/experiment (a minimum of 300 cells). Error bars represent SEM. The asterisk represents significant difference between the mean percent tick cells that internalized beads at the indicated time points. An unpaired, two-tailed t test with Welch’s correction was used to analyze this data. Data were considered significant (*) with p < 0.05.

To determine if the difference in infection rate and levels at 3-hpi and was due to inherent differences in the phagocytic ability of these cell lines, each cell line was inoculated with latex beads and the percent of cells with internalized beads and relative number of internalized beads were compared. The bead internalization was significantly greater in DAE100 compared with ISE6 cultures, with 87% compared to 50% of cells positive for beads (Fig. 5a). Moreover, DAE100 cells contained significantly more beads compared with ISE6 cells (Fig. 5b and c). These results indicate that DAE100 cells have a greater inherent phagocytic ability than ISE6 cells, at least partially accounting for the differences in infection rate between DAE100 and ISE6 cells.

## Discussion

In the United States, *Dermacentor* spp. as a whole, are a primary, epidemiologically relevant vector for *F. tularensis* ssp., though *Amblyomma americanum*, and *Haemaphysallis leporispalustris* are also important vectors in the south central United States^13–16^. The competence of United States populations of *I. scapularis* for *F. tularensis* ssp. has not been experimentally determined, with the exception of an early study by Hopla demonstrating that southern *I. scapularis* could transmit *F. tularensis* to laboratory rabbits^17^. Additionally, in northern Eurasia *I. ricinus* and *I. persulcatus* are competent vectors of *F. tularensis* ssp. *holartica*^18,19^, while *I. ricinus* has been identified as a vector of *F. tularensis* spp. *holarctica* in parts of Europe^20,21^. In contrast, in the northeastern United States, where the epidemiology of *F. tularensis* spp. *tularensis* has been well described, *I. scapularis* has not been implicated as a vector of *F. tularensis* spp. despite its presence in endemic regions^8,22,23^. Further, *I. scapularis* has a broad natural host range that includes rabbits, which are commonly affected by *F. tularensis* spp^24,25^.

In this study, we sought to determine whether the difference in the ecological relevance of *D. andersoni* and *I. scapularis* for *F. tularensis* ssp. transmission in the United States was mirrored at the cellular level. Towards this aim, we determined the competence of two cell lines derived from these tick species, DAE100 and ISE6, to support *F. novicida* infection and replication. We demonstrated the following: i) *F. novicida* can establish infection in DAE100 and ISE6 cells; ii) *F. novicida* infection kinetics differ between DAE100 and ISE6 cells; and, iii) temperature influences bacterial infection kinetics during tick cell infection.

*F. novicida* has a greater capacity to enter and replicates to higher levels in DAE100 cells as compared to ISE6 cells (Fig. 2). The reason for these differences can only be partially explained by the greater cell size and thus increased probability of contacting free bacteria as well as the increased phagocytic capacity of the DAE100 cells^26–28^. The DAE100 cells phagocytosed beads 1.7x more readily than did the ISE6 cells (Fig. 1), whereas the infection rate in the DAE100 cells was 13.6x higher than in the ISE6 cells. Additionally, at 24-hpi *F. novicida* levels declined through time in both cell lines, though host cell death was responsible for this decline only in the DAE100 cells (Fig. 1). Together the increased capacity for *F. novicida* to enter and replicate in DAE100 cells in combination with the reduction in *F. novicida* levels in the absence of cell death in the ISE6 cells indicate differences in pathogen-host cell interaction dependent on the type of host cell. These differences support the utility of different tick cell lines as a tool to study determinants of vector competence.

Important components of vector competence include the ability to invade target tick cells, replicate, and persist within the tick until the tick ingests a second blood meal allowing for transmission of the pathogen to a new host. In these experiments, the reason for the difference in tick cell infection rate is unknown but could be due to inherent differences between the two tick species, including limited expression of key receptor(s) on the surface of tick host cells to initiate entry. The importance of specific tick cell receptors as a determinant of vector competence is highlighted by the requirement of the *I. scapularis* TROSPA midgut protein for *Borrelia burgdorferi* colonization^29^. The ability to replicate and persist in tick cells requires the intracellular pathogen to acquire adequate nutrients and evade immune clearance. The ability of *F. novicida* to replicate to higher levels in the natural vector species could indicate greater availability of nutrients and effective defense mechanisms. In contrast, the reduced numbers of *F. novicida* in ISE6 cells in the absence of cell death may reflect increased clearance by the host cells thus explaining why *I. scapularis* is less associated as a vector for *F. tularensis* ssp.

Although there are strain exceptions, cell damage and death, resulting in decreased vector fitness, is not frequently observed for tick-borne pathogens, as it would be detrimental for transmission. For example, in *D. andersoni* ticks, *F. novicida* reaches a threshold of approximately 1 × 10^7^ CFU/midgut with no overt fitness cost to the tick^10,11^. However, in these studies, *F. novicida* replication lead to significant cell death in the DAE100 cells, indicating a possible lack of signals that limit replication within the tick cells. Consequently, using both *in vivo* and *in vitro* experimental systems are necessary for defining determinants of vector competence^11^.

Temperature is a ubiquitous signal that triggers changes in surface molecule expression, virulence factors and metabolism through altered gene expression^30^. As ticks are ectothermic they must respond to environmental- and host-mediated temperature fluctuations. Therefore, intracellular pathogens must adapt not only to temperature change, but also to the changing environment of the host cell, including altered nutrient availability and cellular responses, as the host cell adapts to the thermal changes. Several tick-borne pathogen studies have demonstrated global transcriptional changes in direct response to temperature alone^31–34^. In this study we examined *F. novicida* infection kinetics at biologically relevant temperatures, 34 °C and 24 °C, representative of tick temperatures during blood feeding and off-host environmental temperatures, respectively. For both cell lines, environmental temperature altered infection kinetics, prolonging elevated bacterial levels and reducing tick cell mortality, particularly in the case of DAE100 cells (Fig. 3). Although identification of the specific mechanisms that allowed for prolonged bacterial levels and reduced tick cell mortality were beyond the scope of this initial study, they are consistent with previous observations that temperature can influence infection success and that intracellular pathogens can dampen virulence to prevent elimination^35,36^.

Towards our goal of understanding determinants of vector competence, we have demonstrated that infection competence differs during *F. novicida* infection of tick cell lines derived from a natural vector and non-vector tick species. Differences in the infection rate, phagocytic capacity, higher bacterial levels in DAE100 cells, and likely clearance of the pathogen in ISE6 cells, contributed to differences in infection competence when comparing these two cell lines. Temperature, the most ubiquitous abiotic factor, affected *F. novicida* infection kinetics and host cell viability similarly in both tick cell lines. The determinants of vector competence are multi-variate and likely highly intertwined. To determine the role of individual determinants will require *in vitro* and *in vivo* infection systems. We propose that *in vitro* and *in vivo F. novicida*-tick infection models provide an ideal opportunity to investigate the cellular and molecular determinants of vector competence for intracellular tick-borne bacterial pathogens. Identifying determinants of vector competence is pivotal to developing effective intervention and control strategies to combat the increasing incidence of tick-borne diseases in humans and animals.

## Materials and Methods

### Bacterial and tick cell culture conditions

Wild-type *F. novicida* strain U112 (generously provided by Dr. Colin Manoil, University of Washington) was used in all experiments. *F. novicida* was cultured in tryptic soy broth (TSB) or on tryptic soy agar (TSA) containing 0.1% L-cysteine as previously described^10^. Briefly, cultures for infection were streaked from −80 °C freezer stocks and grown overnight at 37 °C. *F. novicida* broth cultures were incubated at 37 °C and 225 rpm overnight or for 3 hours (h). *F. novicida* agar cultures were incubated at 34 °C for 24 to 48 h and the resulting colony forming units (CFU) enumerated.

The *D. andersoni* and *I. scapularis* embryonic cell lines DAE100 and ISE6, respectively, were kindly provided by Dr. Ulrike Munderloh at the University of Minnesota and used in all experiments^26,37,38^. Both cell lines were propagated as previously described^38^ and experiments were conducted at 34 °C, unless otherwise noted. For ‘effect of temperature experiments’, tick cell cultures were also incubated at 24 °C.

### *F. novicida*-infection assays

For *F. novicida*-infection assays, tick cells were seeded at 2 × 10^5^ tick cells per well in 24-well plates (Greiner CELLSTAR, Monroe, North Carolina) 12 h prior to *F. novicida*-infection to settle and adhere. The doubling time for both tick cell lines is at least 24 h, so the number of tick cells added to each well was used to calculate the amount of *F. novicida* required for a multiplicity of infection (MOI) of 100. Log-phase *F. novicida* culture was diluted and added to tick cells at an MOI of 100:1 (bacteria:tick cell). To ensure the MOI was accurate, the inoculum was serially diluted and plated on TSA for CFU enumeration. Upon *F. novicida* inoculation, plates were spun at 200 rcf for 5 min to increase proximity of bacteria with the adhered tick cells and then incubated for 2 h at 34 °C in sealed pouches. Media was then removed from wells and cells were washed once with media containing 100 µg/ml gentamicin (Gibco, Grand Island, New York) to remove and kill extracellular bacteria. To each well, 1 ml of fresh media containing gentamicin was added, with the continued gentamicin pressure used to prevent extracellular replication and re-infection of tick cells. Cultures were incubated in sealed pouches and maintained at ambient CO_2_ at 34 °C or 24 °C depending on the experiment. At the designated time-points, media was removed from wells, and cells were lysed by adding 900 µl of sterile water to each well and vigorous pipetting. To the lysed samples, 100 µl of 10X PBS was added and ten-fold serial dilutions of the *F. novicida*-infected cell lysates plated on TSA, incubated at 34 °C for 24 to 48 h and resulting colonies enumerated to determine *F. novicida*-infection levels. Assays were performed simultaneously using DAE100 and ISE6 cells. Uninfected tick cells were used as controls. All assays were repeated at least three times.

### Immunofluorescence microscopy

Immunofluorescence assays (IFA) were used to examine individual tick cell bacterial burdens for each cell line. Tick cells were infected with *F. novicida* as described above with the following modifications: wells were seeded with 1 × 10^6^ tick cells and at 2-hpi samples were washed to remove extracellular bacteria and then re-suspended in media containing 100 µg/ml gentamicin and transferred to chambered well slides (Nunc, Rochester, New York). At 3- or 18-hpi, media was removed and samples were fixed with 4% paraformaldehyde for 10 min. Samples were washed three times with 1X PBS and stained for 1 h using mouse α-*F. novicida* Type 50 (1.5:1000 dilution in 1% BSA, 0.05% Tween20 in 1X PBS) (Creative Diagnostics, Shirley, NY) in the dark at room temperature. Samples were washed three times with 1X PBS and then Alexa Fluor 568-phalloidin (1:500 dilution) and Alexa Fluor 488 goat α-mouse (1:1000 dilution) (Molecular Probes, Eugene, Oregon) was applied for 1 h. Samples were washed three times with 1X PBS and cover-slipped using ProLong Diamond Antifade Mountant with DAPI (Molecular Probes, Eugene, Oregon). Mouse anti-trypanosoma (1:500 dilution) was used as a primary antibody control. Samples were visualized using a Leica TCS SP8 X Confocal Laser Scanning microscope (Leica Microsystems, Bannockburn, IL). The mean proportion of infected tick cells and relative infection level in individually infected cells were manually quantified from six images per assay. Tick cell actin fluorescence was used to determine cell boundaries when counting intracellular bacteria. A minimum of three independent assays were performed for all experiments.

### Phagocytosis assays

Concurrent with *F. novicida*-infection assays for IFA, additional wells of tick cells were exposed to 0.5 µm fluorescent beads (Fluoro-Max, Thermo Scientific, Waltham, Massachusetts), similarly sized to *F. novicida*, to assess the inherent phagocytic characteristic of each tick cell line. Beads were first vortexed for 1 m to create an even suspension and diluted so that tick cells were exposed to 1 × 10^8^ beads per 200 µl, the same concentration and inoculum volume used during bacterial infection. As with *F. novicida* infection, plates were spun at 200 rcf for 5 min to increase contact between beads and adherent tick cells and incubated at 34 °C for 2 h. Wells were washed once with 100 µg/ml gentamicin-containing media and then tick cells were transferred to chambered slides at 2-hours post-bead exposure. Tick cells were allowed to settle for 1 h in chambered slides and were fixed at 3-hours post-bead exposure. Bead-exposed cells were processed in the same manner as *F. novicida*-infected cells for IFA examination of bead internalization.

### Statistical analysis

Statistical analyses were performed using Graphpad Prism 7.01 software. Plating assay CFU counts and trypan blue cell viability counts were compared over multiple time points within and between cell lines using a 1-way ANOVA and Dunnett’s multiple comparisons test or a 2-way ANOVA and Bonferroni’s multiple comparisons test, respectively.

For IFA assays, 6 images from different areas on the slide were used to determine the percent of *F. novicida* infected tick cells and the relative bacterial level of *F. novicida* in individually infected tick cells. For all counts, a minimum of 300 tick cells were examined. The number of infected tick cells and the number of bacteria per cell were quantified from z-stacked images captured using fluorescent microscopy. IFA micrographs were visualized with ImageJ version 1.50j software. To calculate the proportion of tick cells infected with *F. novicida* in each image, we first manually counted the number of DAPI-stained tick cell nuclei at 60X magnification. Within the population of tick cells, both *F. novicida*-infected and uninfected cells were manually tallied. Cell boundaries were determined by using Phalloidin to stain actin. Each infected cell was given a score of ‘1’ based on the presence of *F. novicida* within the cell boundaries. Uninfected tick cells, cells where the presence of *F. novicida* was not detected, were given a score of ‘0’. To determine the infection level of individual infected tick cells, the number of *F. novicida* bacteria were manually counted and grouped as having <5 bacteria or >5 bacteria. Tick cell number and bacterial infection level were enumerated for each sample and the mean counts reported. A similar scoring process was used to quantify the number of tick cells that internalized beads for the phagocytosis assays. The incidence and relative infection level of *F. novicida* infection or bead internalization in individual cells was compared between cell lines or across time points using an unpaired t test with Welch’s correction. For all comparisons, a *P* value of <0.05 was considered significantly different.

### Data availability

All data generated or analyzed during this study are included in this published article (and its Supplementary Information files).

## Electronic supplementary material

Supplemental Figures 1 and 2
