# Supplementary material for: Francisella tularensis novicida infection competence differs in cell lines derived from United States populations of Dermacentor andersoni and Ixodes scapularis
Source: Sci Rep. 2018 Aug 23;8:12685. doi: 10.1038/s41598-018-30419-4 (PMC6107653; doi:10.1038/s41598-018-30419-4)
Supplement: Supplementary file 1 — Supplemental Figures 1 and 2 [file 41598_2018_30419_MOESM1_ESM.docx]

**Full Title:** *Francisella tularensis* *novicida* infection competence differs in cell lines derived from United States populations of *Dermacentor andersoni* and *Ixodes scapularis*

**Authors:** Kathryn E. Reif^1^*, Jessica Ujczo^1^, Debra C. Alperin^2^, Susan M. Noh^1^

**Affiliations:** ^1^Animal Disease Research Unit, Agriculture Research Service, US Department of Agriculture, Pullman, Washington

^2^Department of Veterinary Microbiology and Pathology, Washington State University, Pullman, Washington

**SUPPLEMENTAL FIGURES**


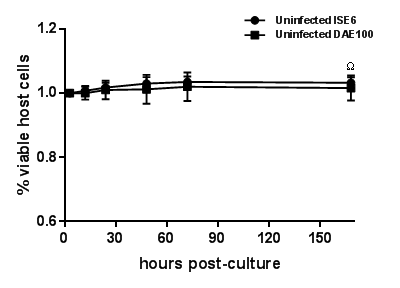


**Supplemental Figure 1. Uninfected tick cell viability.** Tick cell viability was measured for uninfected DAE100 and ISE6 cells over the same experimental timeframe. Tick cell viability (assessed using a trypan blue assay) was quantified by comparing tick cell viability at 12-, 24-, 48-, 72- and 168-hpi to 3-hours post-mock infection. Presented data is the mean of three independent experiments, with each experiment performed in duplicate. Error bars represent SEM. A one-way ANOVA with Dunnett’s correction for multiple comparisons was used to compare viability at different time points within a cell line (^ɸ^represents significant difference in DAE100 cell viability from 3-hpi; ^Ω^represents significant difference in ISE6 cell viability from 3-hpi). Data were considered significant with p < 0.05.


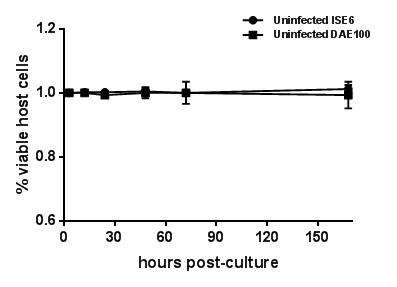


**Supplemental Figure 2. Uninfected tick cell viability at 24°C.** Tick cell viability was measured for uninfected DAE100 and ISE6 cells at 24°C over the same experimental timeframe. Tick cell viability (assessed using a trypan blue assay) was quantified by comparing tick cell viability at 12-, 24-, 48-, 72- and 168-hpi to 3-hours post-mock infection. Presented data is the mean of three independent experiments, with each experiment performed in duplicate. Error bars represent SEM. A one-way ANOVA with Dunnett’s correction for multiple comparisons was used to compare viability at different time points within a cell line (^ɸ^represents significant difference in DAE100 cell viability from 3-hpi; ^Ω^represents significant difference in ISE6 cell viability from 3-hpi). Data were considered significant with p < 0.05.
